# Characterization of a NDM-1- Encoding Plasmid pHFK418-NDM From a Clinical *Proteus mirabilis* Isolate Harboring Two Novel Transposons, Tn*6624* and Tn*6625*

**DOI:** 10.3389/fmicb.2019.02030

**Published:** 2019-09-04

**Authors:** Dandan Dong, Manli Li, Zhenzhen Liu, Jiantao Feng, Nan Jia, Hui Zhao, Baohua Zhao, Tingting Zhou, Xianglilan Zhang, Yigang Tong, Yuanqi Zhu

**Affiliations:** ^1^Department of Laboratory Medicine, The Affiliated Hospital of Qingdao University, Qingdao, China; ^2^Department of Laboratory Diagnostics, The Medical Faculty of Qingdao University, Qingdao, China; ^3^State Key Laboratory of Pathogen and Biosecurity, Beijing Institute of Microbiology and Epidemiology, Beijing, China; ^4^College of Life Science, Hebei Normal University, Shijiazhuang, China; ^5^College of Information Science and Technology, Beijing University of Chemical Technology, Beijing, China

**Keywords:** *Proteus mirabilis*, *bla*_NDM–__1_, transposons, plasmids, multidrug-resistant

## Abstract

Acquisition of the *bla*_NDM–__1_ gene by *Proteus mirabilis* is a concern because it already has intrinsic resistance to polymyxin E and tigecycline antibiotics. Here, we describe a *P. mirabili*s isolate that carries a pPrY2001-like plasmid (pHFK418-NDM) containing a *bla*_NDM–__1_ gene. The pPrY2001-like plasmid, pHFK418-NDM, was first reported in China. The pHFK418-NDM plasmid was sequenced using a hybrid approach based on Illumina and MinION platforms. The sequence of pHFK418-NDM was compared with those of the six other pPrY2001-like plasmids deposited in GenBank. We found that the multidrug-resistance encoding region of pHFK418-NDM contains ΔTn*10* and a novel transposon Tn*6625*. Tn*6625* consists of ΔTn*1696*, Tn*6260*, In251, ΔTn*125* (carrying *bla*_NDM–__1_), ΔTn*2670*, and a novel *mph*(E)-harboring transposon Tn*6624*. In251 was first identified in a clinical isolate, suggesting that it has been transferred efficiently from environmental organisms to clinical isolates. Genomic comparisons of all these pPrY2001-like plasmids showed that their relatively conserved backbones could integrate the numerous and various accessory modules carrying multifarious antibiotic resistance genes. Our results provide a greater depth of insight into the horizontal transfer of resistance genes and add interpretive value to the genomic diversity and evolution of pPrY2001-like plasmids.

## Introduction

Urinary tract infections (UTIs) are the most common bacterial infections (Gastmeier et al., 1998). Cases of UTIs can be classified as uncomplicated or complicated (Beahm et al., 2017). Clinically, *Proteus mirabilis* is most frequently a pathogen of UTIs, particularly in patients suffering from complicated cUTIs (Schaffer and Pearson, 2015). Although *Escherichia coli* is the primary urinary tract pathogen, *P. mirabilis* ranks third as the cause of UTIs and accounts for 4.1% of urinary tract infection isolates in CANWARD surveillance study, 4.6% in southern China, respectively (Karlowsky et al., 2011; Li et al., 2017). Because this pathogen is intrinsically resistant to nitrofurantoin, polymyxin, and tigecycline antibiotics (Ramos et al., 2018), acquiring additional carbapenemase antibiotic resistance is worrisome (Reffert and Smith, 2014). Currently, fosfomycin, which is previously used mainly as oral treatment for UTIs, has gained clinicians’ attention worldwide because of its activity against multidrug-resistant bacteria (Reffert and Smith, 2014; Giske, 2015). In additionally, fosfomycin resistance rates are generally low but substantially higher when carbapenemase producers are considered (Giske, 2015). One such resistance gene is *bla*_NDM–__1_ (New Delhi metallo-β-lactamase), which was initially identified in a *Klebsiella pneumoniae* strain (Yong et al., 2009). Isolates of this species that harbor the *bla*_NDM–__1_ gene can hydrolyze nearly all β-lactam antibiotics except aztreonam. Therefore, the acquisition of *bla*_NDM–__1_ by *P. mirabilis* would be problematic, as it would greatly reduce the therapeutic options for treating infections caused by it.

The *bla*_NDM–__1_ gene is mainly and widely spread by an IS*Aba125*-bounded composite transposon Tn*125* (Poirel et al., 2012; Ranjan et al., 2016), and *bla*_NDM–__1_-carrying plasmids are commonly found in IncA (Solgi et al., 2017), IncC (Harmer and Hall, 2017), IncT (Mataseje et al., 2016), IncR (Gamal et al., 2016), IncFII (Lin et al., 2016), IncX (Wang et al., 2018), and IncN (Wang et al., 2018) incompatible groups. However, *bla*_NDM–__1_-carrying plasmids have gradually appeared in some unknown incompatibility groups. The *bla*_NDM–__1_-harboring pHFK418-NDM plasmid and six other plasmids have been assigned into the same unknown incompatibility group based on their replicons. The six plasmids are pPrY2001 (Accession no. KF295828) (Mataseje et al., 2014), p06-1619-1 (Accession no. KX832929) (Marquez-Ortiz et al., 2017), p16Pre36-NDM (Accession no. KX832927) (Marquez-Ortiz et al., 2017), pPp47 (Accession no. MG516912) (Dolejska et al., 2018), pPm60 (Accession no. MG516911) (Dolejska et al., 2018), and pC131 (Accession no. KX774387). The earliest reported plasmid, pPrY2001, is considered to be the reference plasmid, so the above-named plasmids are called pPrY2001-like plasmids (Marquez-Ortiz et al., 2017; Dolejska et al., 2018). Up to now, no studies in the published scientific literature have thoroughly analyzed and compared in detail the structures and genomes of this unknown incompatibility group.

Here, we studied the *bla*_NDM–__1_-harboring plasmid, pHFK418-NDM, a known pPrY2001-like plasmid according to its replicon, which was first isolated from a clinical *P. mirabili*s HFK418 strain in China. We elucidated the complete sequence of pHFK418-NDM (which carries two novel transposons, Tn*662*4 and Tn*6625*) and compared it with six other pPrY2001-like plasmids to obtain insight into the horizontal transfer of resistance genes and the diversity and evolution of pPrY2001-like plasmids.

## Materials and Methods

### Species Identification and Antimicrobial Susceptibility Testing

The study was approved by the Medical Ethics Committee at the Affiliated Hospital of Qingdao University, China, and written informed consent was received from the patient. The *P. mirabilis* HFK418 strain was isolated from the urine specimen of a patient with epidemic encephalitis at the Affiliated Hospital of Qingdao University, China, in 2017. Referring to the method described in Ranjan et al. (2016), this strain was multiple tested for purity by routine laboratory methods, then the pure strain was cryopreserved at −80°C in 50% glycerol. The pure isolate was revived in Luria-Bertani (LB) broth (BD Biosciences, United States) with 4 μg/ml meropenem to experiments. The *P. mirabilis* HFK418 isolate was identified and subjected to antimicrobial susceptibility testing using the VITEK compact-2 automated system (bioMérieux, France). In addition, fosfomycin MICs were further determined by fosfomycin *E*-tests (bioMérieux). CLSI (Clinical and Laboratory Standards Institute) 2018 breakpoints were used (M100-S28) (CLSI, 2018).

### Antimicrobial Resistance Gene Screening and Plasmid Conjugal Transfer

The major acquired extended-spectrum β-lactamase (Dallenne et al., 2010; Hussain et al., 2014; Ranjan et al., 2017), fosfomycin (Dantas Palmeira et al., 2018), chloramphenicol (White et al., 1999), lincosamide (Garcia-Martin et al., 2018), and carbapenemase genes (Chen et al., 2015; Ranjan et al., 2016, 2017) were detected by PCR, after which all the PCR amplicons were sequenced on the ABI 3730 platform (Applied Biosystems, United States). The sodium azide-resistant *E. coli* J53Azi^R^ strain was used as the recipient and the *P. mirabilis* HFK418 isolate as the donor for the conjugative transfer of the plasmids. The conjugal transfer tests were performed as described previously (Srijan et al., 2018), and the conjugation frequency was calculated as transconjugants divided by number of donors.

### Carbapenemase Activity Assay

To determine whether the *bla*_NDM–__1_ gene was expressed in both *P. mirabilis* HFK418 and the *E. coli* J53Azi^R^ transconjugant HFK418-NDM-J53 strain, we performed an imipenem–EDTA *E*-test (AB-BioMérieux, Sweden) to assess the class B carbapenemase activity.

### Sequencing and Sequence Assembly

Bacterial genomic DNA was extracted using the Wizard Genomic DNA Purification Kit (Promega, United States), followed by the MiSeq (Illumina, United States) and MinION (Oxford Nanopore, United Kingdom) sequencing. The short Illumina reads were trimmed to remove the poor quality sequences, and the resultant contigs were assembled using Newbler3.0 (Nederbragt, 2014). The longest single read obtained by the MinION sequencer was 98 kb, thereby crossing the repetitive shufflon regions in the plasmid (Laver et al., 2015). The long reads from the MinION combined with the short Illumina reads were hybrid assembled using SPAdesv3.11.1 (Bankevich et al., 2012). Hybrid assembly produced several scaffolds and BLASTN analysis confirmed that the scaffold in our study has the highest similarity to the plasmid p16Pre36-NDM (Accession no. KX832927) with coverage of 69% and identity of 96%. As most of the published plasmids are in a circle form, further bioinformatics analysis confirmed that this scaffold can be successfully cyclized using our in-house script. The correctness was then proved by mapping the high-throughput sequencing reads to the cyclized scaffold using CLC Genomics Workbench 9.0, with a mean reads mapping coverage of 111x. The consensus sequence acquired from CLC Genomics Workbench 9.0 was finally treated as the complete sequence of our plasmid pHFK418-NDM.

### Sequence Annotation and Genome Comparisons

Open reading frames (ORFs) and pseudogenes that were predicted by RAST2.0 (Brettin et al., 2015) were further annotated using BLASTP/BLASTN (Boratyn et al., 2013) against the RefSeq databases (O’Leary et al., 2016) and UniProtKB/Swiss-Prot (Boutet et al., 2016). Mobile elements, resistance genes, and other features were annotated by INTEGRALL (Moura et al., 2009), ISfinder (Siguier et al., 2006), ResFinder (Kleinheinz et al., 2014), PlasmidFinder (Carattoli et al., 2014), and the Tn Number Registry (Roberts et al., 2008) online databases. Comparisons of the multiple and paired sequences were conducted using MUSCLE 3.8.31 and BLASTN, respectively. Gene organization diagrams were drawn in Inkscape0.48.1^1^.

### Nucleotide Sequence Accession Number

The complete nucleotide sequence of plasmid pHFK418-NDM has been deposited in the National Center for Biotechnology Information nucleotide database^2^ under accession number MH491967.

## Results and Discussion

### Characterization of *P. mirabilis* HFK418

Plasmid pHFK418-NDM from *P. mirabilis* HFK418 was transferable to *E. coli* J53Azi^R^ in the conjugation experiments, thereby generating the *bla*_NDM–__1_-positive *E. coli* J53Azi^R^ transconjugant HFK418-NDM-J53 strain. The conjugation frequency was 1.5 × 10^–2^.

Imipenem-EDTA E-tests were positive in both *P. mirabilis* HFK418 and HFK418-NDM-J53. These two strains were highly resistant to ampicillin, cefazolin, cefuroxime, ceftazidime, ceftriaxone, imipenem, and meropenem, but not to aztreonam, revealing that pHFK418-NDM is a conjugative NDM-encoding plasmid with carbapenemase activity (Table 1 and Supplementary Figure S1).

**TABLE 1:** Antimicrobial susceptibility profiles.

**Antibiotics**	**MIC (mg/L)/antimicrobial susceptibility^∗^**		
	
	**HFK418**	**HFK418**-**NDM**-**J53**	**J53**
Ampicillin	≥32/R	≥32/R	=8/S
Cefazolin	≥64/R	=32/R	≥4/S
Cefuroxime	≥64/R	≥64/R	=4/S
Ceftazidime	≥64/R	≥64/R	≤1/S
Ceftriaxone	≥64/R	≥64/R	≤1/S
Imipenem	≥16/R	≥16/R	≤1/S
Meropenem	≥16/R	≥16/R	≤0.25/S
Aztreonam	≤1/S	≤1/S	≤1/S
Gentamicin	≥16/R	=2/S	≤1/S
Ciprofloxacin	≥4/R	≤0.25/S	≤0.25/S
Levofloxacin	≥8/R	≤0.25/S	≤0.25/S
Fosfomycin	≥1024/R	=4/S	=2/S
Nitrofurantin	≥512/R	=64/I	≤16/S
Trimethoprim/sulfamethoxazole	≥320/R	=40/S	≤20/S

*^∗^The interpretation is derived from the Clinical and Laboratory Standards Institute Guidelines (CLSI, 2018) (S, sensitive; R, resistant; I, intermediately resistant).*

### Overview of Plasmid pHFK418-NDM

PCR screening for antimicrobial resistance genes showed that *P. mirabilis* HFK418 carries *bla*_NDM–__1_, *bla*_OXA–__1_, *bla*_CTX–M–__65_, *fosA3*, *catB5*, *lnu*(G), and *bla*_OXA–__10_ genes. The complete sequence of pHFK418-NDM is 145,619 bp with a mean G + C content of 42.8%, and 157 ORFs (Table 2 and Supplementary Figure S2). Based on the replicon, pHFK418-NDM was assigned into the unknown incompatibility group of pPrY2001-like plasmids. The linear genomic comparison conducted between pHFK418-NDM and six other pPrY2001-like plasmids [pPrY2001 (Mataseje et al., 2014), p06-1619-1 (Marquez-Ortiz et al., 2017), pC131, pPp47 (Dolejska et al., 2018), pPm60 (Dolejska et al., 2018), and p16Pre36-NDM (Marquez-Ortiz et al., 2017)] showed that the highest sequence homology belonged to pHFK418-NDM with >69% query coverage and >99% identity (Supplementary Figure S3 and Supplementary Data Sheet S2).

**TABLE 2:** Major features of pPrY2001-like plasmids in this work.

**Category**	**pPrY2001-like plasmids**						
	
	**pPrY2001**	**p06-1619-1**	**pC131**	**pHFK418-NDM**	**pPp47**	**pPm60**	**p16Pre36-NDM**
Accession number	KF295828	KX83299	KX77437	This study	MG516912	MG516911	KX83297
Strain	*P. rettgeri*	*P. rettgeri*	*P. rettgeri*	*P. mirabilis*	*P. mirabilis*	*P. mirabilis*	*P. rettgeri*
Source	Clinical	Clinical	Clinical	Clinical	Wildlife	Wildlife	Clinical
Country	Canada	American	Brazil	China	Australia	Australia	American
Total length(bp)	113, 295	90, 666	118, 501	145, 619	142, 085	113, 297	244, 116
Total number of ORFs	123	97	125	157	161	127	270
Mean G + C content,%	41.3	37.5	40.8	42.8	42.7	40.9	47.9
Length of the backbone (bp)	74, 670	72, 067	77, 414	69, 823	69, 543	68, 879	150, 505

The genomic structures of the pPrY2001-like plasmids comprised two major regions: the backbone and accessory module. The backbone could be further divided into three parts: the replication genes (*repA* and its iterons), the conjugal transfer genes (*tiv*, *rlx*, and *cpl*), and the plasmid maintenance genes (*parFG*, *MazFE*, *stbB*, *ssb*, and *flhC*). Each plasmid’s backbone was able to integrate two or more accessory modules by transposition or recombination events. pHFK418-NDM contains two accessory modules, the Tn*6901* related region and the multidrug-resistant (MDR) region, while the MDR region contains Tn*6625* and ΔTn*10* (Supplementary Figures S2, S3).

### Backbone Regions in the pPrY2001-Like Plasmids

Our pairwise comparison analysis of the pPrY2001-like plasmids backbones showed that they shared >96% nucleotide identity across >42%, indicating that their backbones were relatively conserved. However, there were three major differences among all their backbones. (I) the *parC* gene (centromere, binding sites for *parG*) did not exist in pPrY2001, and the copy numbers of the 8-bp tandem repeat (TGTGTata) within the *parC* gene varied among the other plasmids (4 for p06-1619-1, pC131, and pPm60; 5 for pPp47, pHFK418-NDM, and p16Pre36-NDM). (II) Compared with the conjugal transfer region in the other plasmids, the *rlx* gene from pPrY2001 is disrupted into Δ*rlx-*3′ and Δ*rlx-*5′ by insertion of IS*Prre5* (named in this study). (III) The hybrid backbone of plasmid p16Pre36-NDM was acquired from a pPrY2001-like plasmid and the IncC2 plasmid (the *orf1847* and *rhs2* marked genes) (Supplementary Figure S3).

### The MDR Region Harbors the *bla*_NDM–__1_ Gene From pPrY2001-Like Plasmids

**FIGURE 1:**
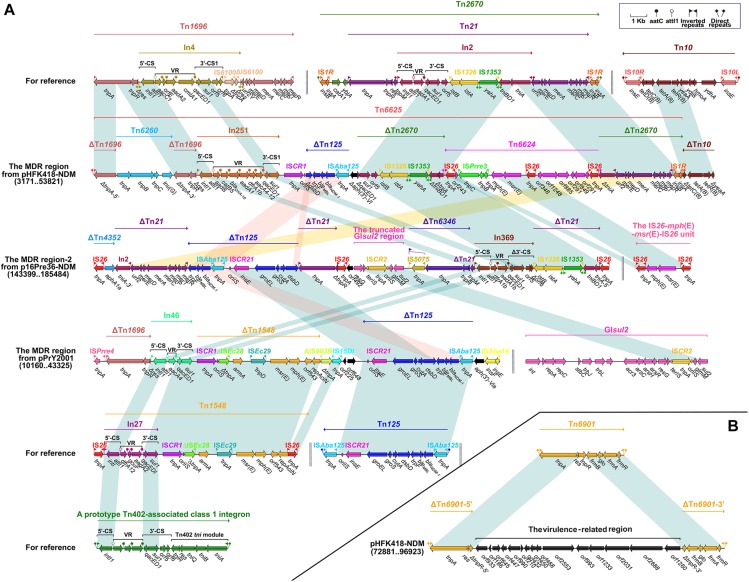
The MDR region harboring *bla*_NDM–1_ of pPrY2001-like plasmids and comparison with related regions **(A)**. Accessory module outside the MDR region of pHFK418-NDM **(B)**. Genes, mobile elements and other features are colored based on function classification. Numbers in parentheses show the nucleotide positions within the corresponding plasmids. Shading regions denote shared DNA regions of homology (>95% nucleotide identity). MDR: multidrug-resistant.

We found that the *bla*_NDM–__1_-carrying ΔTn*125* transposon is present in the MDR region of pHFK418-NDM, p16Pre36-NDM (the MDR region-2), and pPrY2001. Tn*125*, an IS*Aba125*-bounded composite transposon in plasmid pNDM-BJ01, was acquired from *Acinetobacter lwoffii* (Poirel et al., 2012). It is made up of IS*Aba125*, *bla*_NDM–__1_, *ble*_MBL_ (bleomycin resistance), t*rpF*, *dsbD*, *cutA*, *groES*, *groEL* and IS*CR21*, and is bordered by 3-bp direct repeats (DRs: target site duplication signals for transposition). In the MDR region of these three plasmids, ΔTn*125* has undergone the deletion of IS*Aba125* downstream of IS*CR27*. In addition, ΔTn*125* from pHFK418-NDM and p16Pre36-NDM contain the following differences: ΔTn*125* in pHFK418-NDM has a Δ*dsbD*–t*rpF*–*ble*_MBL_–*bla*_NDM–__1_–IS*Aba125* structure, while the IS*CR21*–*groEL*–*groES*–*cutA*–*dsbD* fragment, which occurs upstream of *bla*_NDM–__1_ in p16Pre36-NDM, was generated by complex recombination events (Figure 1A).

**FIGURE 2:**
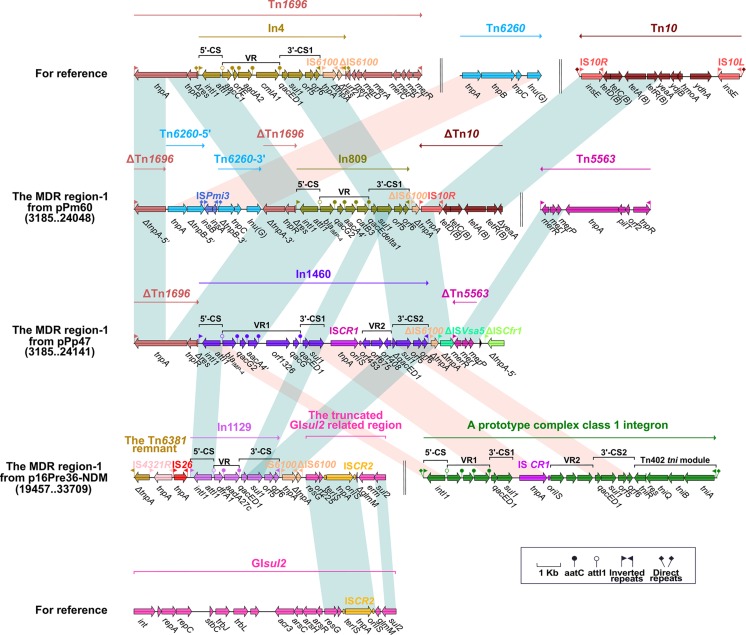
The MDR region carrying *bla*_IMP–4_ and comparison with related regions. Genes are denoted by arrows. Mobile elements, genes, and other features are colored based on function classification. Numbers in parentheses denote the nucleotide positions within the corresponding plasmids. Shading regions show shared DNA regions of homology (>95% nucleotide identity). MDR: multidrug-resistant.

Integron In251, which is located upstream of ΔTn*125* in pHFK418-NDM, belongs to the prototypic Tn*402*-associated class 1 integron. This class 1 integron can be divided sequentially into an IRi (inverted repeat at the integrase end), a 5′-conserved segment (5′-CS: *intI1*–*attI1*), a variable region (VR: containing one or more resistant genes), a 3′-conserved segment (3’-CS: *qacED1*–*sul1*–*orf5*–*orf6*), the Tn*402 tni* module (*tniA*–*tniB*–*tniQ*–*res*–*tniR*) and IRt (inverted repeat at the *tni* end), and is surrounded by 5-bp DRs. Furthermore, In369 (in MDR region-2 from p16Pre36-NDM), In46 (in the MDR region from pPrY2001), In809 (in the MDR region-1 from pPm60), and In1129 (in the MDR region-1 from p16Pre36-NDM) are also different derivatives from the prototypical Tn*402*-associated class 1 integron. The structures of In251, In369, and In46 are arranged as IRi, 5′-CS, VR (*aadB*–*catB5*–*bla*_OXA–__10_–*aadA1*–*dfrA1*–*aacA4-12* in In251, *dfrA1b*–*aadA1b* in In369, and *aacA4* in In46), and Δ3′-CS (*qacED1*–*sulI* in In251and In46, *qacED1*–*sulI*–*orf5*–Δ*orf6* in In369), without the Tn*402 tni* module and IRt. The Tn*402 tni* module and IRt have been replaced downstream by other mobile elements. In809 and In1129 each have the following common structure: IRi, 5′-CS, VR, 3′-CS, and IRt, and their Tn*402 tni* module has been lost during the evolutionary process. A difference between In809 and In1129 is apparent in the variable region (*dfrA1*–*aadA27c* in In1129, *bla*_IMP–__4_–*qacG2*–*aacA4*′–*catB3* in In809). ΔTn*1696* is embedded upstream of the class 1 integrons In251, In46, In809, and In1460 (in the MDR region-1 from pPp47). The Tn*1696* prototype comprises an IRL (inverted repeat left)–*tnpA* (transposase)–*tnpR* (resolvase)–*res* (resolution site)–*mer* (mercury resistance)–IRR (inverted repeat right) structure, and a *res* site is interrupted by insertion of In4 into 75-bp Δ*res-*5′ and 45-bp Δ*res-*3′. Compared with the structure of Tn*1696*, ΔTn*1696* has the same IRL–*tnpA*–*tnpR*–Δ*res-*5′ module in the MDR region of pHFK418-NDM, pPrY2001, pPm60, and pPp47. The ΔTn*1696 tnpA* from pHFK418-NDM and pPm60 is segmented into two fragments, Δ*tnpA-*5′ and Δ*tnpA-*3′, by insertion of Tn*6260*. Belonging to the Tn*554* family, Tn*6260* consists of *tnpA*, *tnpB*, *tnpC*, and *lnu*(G) (lincosamide resistance), as identified in *Enterococcus thailandicus* a523 (Ybazeta et al., 2017), *Virgibacillus halodenitrificans* PDB-F2 (Tao et al., 2016), and *E. faecalis* E531 (Zhu et al., 2017). Up until now, Tn*6260* only appeared in pPrY2001-like plasmids when pHFK418-NDM and pPm60 were present. Moreover, IS*Pmi3* split *tnpB* of Tn*6260* from pPm60 into two parts, Δ*tnpB-*5′ and Δ*tnpB-*3′, which are surrounded by 8-bp DRs (Figures 1A, 2).

ΔTn*2670* from pHFK418-NDM is integrated downstream of ΔTn*125*. Flanked by 9-bp DRs, Tn*2670* is organized as IS*1R*, *catA1* (chloramphenicol resistance), *ybjA* (acetyl transferase), Tn*21*, and IS*1R*, and was initially discovered in plasmid R100 from *Shigella flexneri* (Partridge and Hall, 2004). Tn*21*, a Tn*3*-family transposon unit, contains an IRL–*tnpA*–*tnpR*–*res*–*tnpM* (modulator protein)–In2–*urf2*–the *mer operon*–IRR module, and a presumed ancestral *urf2M* gene is interrupted by insertion of In2 to generate *tnpM* and *urf2* (Liebert et al., 1999). In2 comprises IRi, 5′-CS, VR (*aadA1*), 3′-CS, IS*1326*, IS*1353*, the *tni* module, and IRt, and is delimited by 5-bp DRs. In terms of the structure of Tn*2670*, ΔTn*21* can be divided into four segments in the MDR region from p16Pre36-NDM; namely, (I) IRL, *tnpA*, and Δ*tnpR*, (II) *tnpM*, (III), In2 (IS*1326*, IS*1353*, the disrupted *tni* module), and (IV), In2 (the disrupted *tni* module and IRt), *urf2*, the *mer* operon, and IRR. These four segments fall within different positions by virtue of transposition or recombination events. In pHFK418-NDM, ΔTn*2670* reserves a fragment from the 3′-CS of In2 to IS*1R*, but its *tniA* gene is segmented into two fragments (Δ*tniA*_In__2_-5′ and Δ*tniA*_In__2_-3′) by insertion of Tn*6624* (Figure 1A).

Tn*6624*, a novel IS*26*-based transposon unit, has been inserted into the pHFK418-NDM plasmid from *P. mirabilis* HFK418. Delimited by 8-bp DRs (CATCGGCG), it has the following mosaic structure: IS*26*, a novel IS*66*-family IS*Prre3*, *mph*(E) (macrolide resistance), *msr*(E) (macrolide efflux protein), IS*26*, a fragment with an unknown function, and IS*26*. The *mph*(E)–*msr*(E)–IS*26* fragment originated from the IS*26*–*mph*(E)–*msr*(E)–IS*26* transposon unit and was initially identified in the chromosomal integrative conjugative element from *Pasteurella multocida* (Michael et al., 2012). Three copies of IS*26* are present in Tn*6624*, which promotes the formation and transposition of Tn*6624*. Another novel 48,068 bp multidrug resistance transposon, Tn*6625*, was found in the pHFK418-NDM plasmid from *P. mirabilis* HFK418. The ΔTn*1696*, Tn*6260*, In251, ΔTn*125*, Tn*6624*, and ΔTn*2670* mobile elements have been described in detail above, and all of them are included in the large composite Tn*6625* transposon. Tn*6625* carries twelve resistance genes, bounded by 3-bp DRs (TTG). Tn*6625* contains integron In25, which has so far only been found in wastewater-isolated *Providencia* VIGAT3 (Guo et al., 2011). Thus, In251 was first isolated from clinical *P. mirabilis* HFK418, suggesting that it has been efficiently transferred from environmental micro-organisms to clinical isolates (Figure 1A).

**FIGURE 3:**
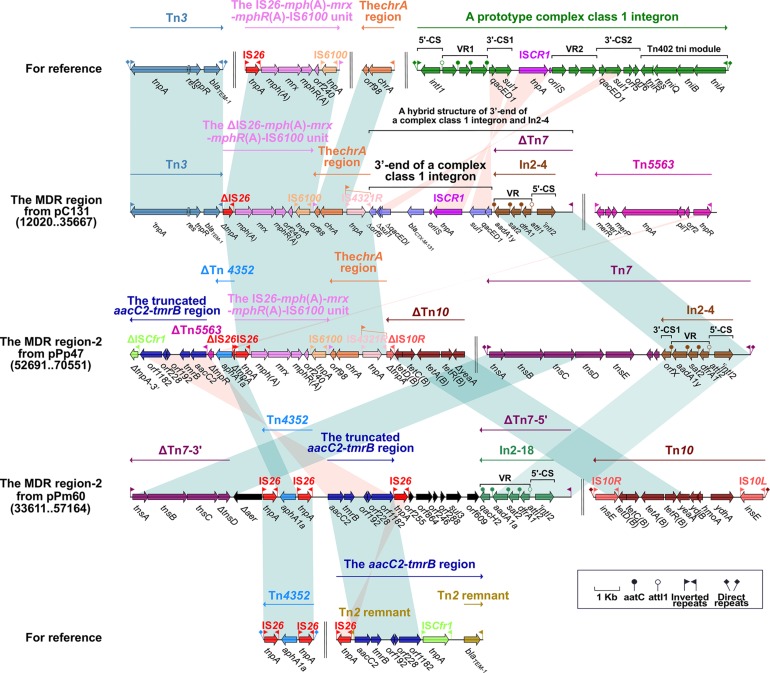
The MDR region harboring class 2 integron and comparison with related regions. Genes are denoted by arrows. Genes, mobile elements and other features are colored based on function classification. Numbers in parentheses denote the nucleotide positions within the corresponding plasmids. Shading regions indicate shared DNA regions of homology (>95% nucleotide identity). MDR: multidrug-resistant.

The MDR region of pHFK418-NDM includes Tn*6625* and ΔTn*10.* Delimited by 9-bp DRs, Tn*10* is arranged sequentially as IS*10L*, *ydhA*, *hmoA*, *ydjB*, *yeaA*, *tetR*, *tetA* (tetracycline resistance), *tetC*, *tetD*, and IS*10R*, as identified in the conjugative R27 plasmid from *Salmonella typhi* (Lawley et al., 2000). ΔTn*10* was found in the MDR region of pHFK418-NDM, pPp47, and pPm60, and comprises a common fragment (*tetD*–*tetC*–*tetA*–*tetR*–Δ*yeaA*). But IS*10R* is absent in pHFK418-NDM, truncated in pPp47, and intact in pPm60. Tn*10* is also integrated between *orf153* and *orf489* in the backbone of p16Pre36-NDM, bracketed by 9-bp DRs. Tn*10* is an integral transposon in p16Pre36-NDM, but its IS*10R* has two segments (ΔIS*10R-*5′ and ΔIS*10R-*3′) and is disrupted by insertion of IS*Kpn26* with 4-bp DRs (Figures 1A, 2, 3).

There are other transposon units also (ΔTn*6346*, the truncated GI*sul2* region, and ΔTn*1548*) in the MDR region of p16Pre36-NDM and pPrY2001, except as described above. ΔTn*6346* and the truncated GI*sul2* region are embedded in the MDR region of p16Pre36-NDM. Tn*6346*, a Tn*3*-family transposon, was discovered in heavy metal-tolerant *Achromobacter* AO22 (Ng et al., 2009). In the MDR region-2 of p16Pre36-NDM, ΔTn*6346* is arranged in turn as the IS*5075* interrupted-IRL, *tnpA*, *tnpR*, and 121-bp Δ*res*, and the lost *mer* operon and IRR were replaced by *tnpM* from ΔTn*21*. GI*sul2* is arranged sequentially as *int* (integrase), several conjugation transfer genes, *resG* (resolvase), IS*CR2*, *glmM* (phosphoglucosamine mutase) and *sul2*, which are found in various bacterial species (Nigro and Hall, 2011). In the MDR region-2 from p16Pre36-NDM, ΔGI*sul2* comprises *resG*, *orf225*, IS*CR2*, *glmM*, and *sul2*. In the MDR region-1 of p16Pre36-NDM, the truncated GI*sul2* related region has a *resG*–*orf225*–IS*CR2*–Δ*glmM*–*erm* (rRNA adenine N-6-methyltransferase)–*sul2* structure. The *erm* resistance gene is present 100-bp downstream of IS*CR2*. The truncation of *glmM* and the appearance of *erm* are correlated with IS*CR2*-mediated transposition. ΔTn*1548* is present in the MDR region of pPrY2001, and Tn*1548* was initially discovered in plasmid pCTX-M3 from *Citrobacter freundii* (Dolejska et al., 2013). Compared with the structure of Tn*1548*, ΔTn*1548* comprises IS*CR1*, ΔIS*Ec28*, *armA* (aminoglycoside resistance), IS*Ec29*, *msr*(E), *mph*(E), *orf543*, and Δ*repAci* (Figure 1A).

### The MDR Region Harbors the *bla*_IMP–__4_ Gene From pPrY2001-Like Plasmids

The *bla*_IMP–__4_ gene is integrated into the integron (In809 and In1460) of the MDR region-1 from pPm60 and pPp47. In809 is a prototype Tn*402*-associated class 1 integron, and its VR region includes *bla*_IMP–__4_, *qacG2*, *aacA4*′, and *catB3*. In1460 is a complex class 1 integron made up of IRi, 5′-CS, VR1 (variable region 1), 3′-CS1 (*qacED1*–*sul1*), IS*CR1* (common region), VR2 (variable region 2), 3′-CS2 (*qacED1*–*sul1*–*orf5*–*orf6*), the Tn*402 tni* module and IRt, bounded by 5-bp DRs. In1460 comprises IRi, 5′-CS, VR1 (*bla*_IMP–__4_–*qacG2*–*aacA4*′–*orf1326*–*qacG*), 3′-CS1 (*qacED1*–*sul1*), IS*CR1*, VR2 (Δ*orf453*–*orf675*–*orf408*), 3′-CS2 (Δ*qacED1*–*sul1*–*orf5*–*orf6*), and IRt. The insertion of VR2 between IS*CR1* and 3′-CS2 truncates *qacED1* at the 5′ terminal of the 3′-CS2 (Figure 2).

### The MDR Region Harbors a Class 2 Integron From pPrY2001-Like Plasmids

In2-4 (in the MDR region from pC131) and In2-18 (in the MDR region-2 from pPm60) can be classified as class 2 integrons, embedded in ΔTn*7*. The class 2 integron is found in transposon Tn*7* and its derivatives (Hansson et al., 2002). Bracketed by 5-bp DRs, Tn*7* contains IRL–In2-4–the *tns* module (*tnsE*–*tnsD*–*tnsC*–*tnsB*–*tnsA*)–IRR (Peters, 2014). Integron In2-4 contains 5′-CS (*intI2*–*attI2*), VR (*dfrA* [dihydrofolate reductase]–*sat2* [streptothricin acetyltransferase]–*aadA1y* [aminoglycoside adenylyltransferase]), and 3′-CS (*ybeA*, also known as *orfX*) (Hansson et al., 2002). ΔTn*7* from pC131 has a core module (from IRL to the VR of In2-4), and its missing portion has been replaced by the 3’-end of a complex class 1 integron. The 3′ end of the complex class 1 integron includes 3′-CS1, IS*CR1*, VR2 (*bla*_CTX–M–__131_), and Δ3′-CS2 (Δ*qacED1*–Δ*sul1*–Δ*orf5*); the truncated 3’-CS2 results from its positioning between VR2 and the *chrA* region. It is apparent that ΔTn*7* from pPm60 is arranged as an IRL, In2-18 (5′-CS and VR [*dfrA1*–*sat2*–*aadA1a*–*qacH2*]), a truncated *tns* module (Δ*tnsD*–*tnsC*–*tnsB*–*tnsA*), and an IRR. Insertion of the accessory region (from Δ*aer* to *orf609*) means that ΔTn*7* is split into two separate portions; namely, ΔTn*7*-5′ and ΔTn*7*-3′ (Figure 3).

The transposon unit IS*26*–*mph*(A)–*mrx*–*mphR*(A)–IS*6100* and the *chrA* region were found to be inserted into the upstream region of the 3’-end of a complex class 1 integron in pC131. The macrolide resistance unit IS*26*–*mph*(A)–*mrx*–*mphR*(A)–IS*6100* is considered to be a mobile element, and the *mph*(A)–*mrx*–*mphR*(A) operon encodes a phosphotransferase, a positive regulator factor, and a negative transcription factor (Partridge, 2011). This transposon unit is truncated in the MDR region of pC131, but it is present as an intact structure in the MDR region-2 of pPp47, while in pC131, the transposon unit (ΔIS*26*–*mph*(A)–*mrx*–*mphR*(A)–IS*6100*) is situated between Tn*3* and the *chrA* region. Tn*3* carries the class A beta-lactamase-encoding *bla*_TEM–__1_ gene, which was initially observed as an R1 plasmid in *E. coli* (Bailey et al., 2011). Here, Tn*3* is an unabridged transposon in pC131, but its *tnpA* is a pseudogene. The *chrA* region (IRL_chrA_–*chrA* [chromate resistance]–*orf98*) is derived from a Tn*21*-like transposon in plasmid pCNB1 from *Comamonas*, and is often closely linked to IRt–IS*6100* (Partridge, 2011). The *chrA* region, which is connected with the IS*26*–*mph*(A)–*mrx*–*mphR*(A)–IS*6100* unit in pC131 and pPp47, has arisen through IS*6100*-mediated recombination. The *chrA* region includes the IS*4321R* interrupted-IRL_chrA_, *chrA* and *orf98* in pC131 and pPp47. Insertion of IS*4321R*, IRL_chrA_ is disrupted and forms two parts, ΔIRL_chrA_-5′ and ΔIRL_chrA_-3′ (Figure 3).

We found that Tn*4352* and the truncated *aacC2*–*tmrB* region are integrated between ΔTn*7*-3′ and ΔTn*7*-5′ in pPm60. Flanked by 8-bp DRs at both ends, Tn*4352* is an IS*26*-bounded structure (IS*26*–*aphA1a*–IS*26*), and the *aphA1a* resistance gene confers resistance to kanamycin and neomycin (Wrighton and Strike, 1987). Although Tn*4352* is complete in the MDR region-2 from pPm60, it is truncated in the MDR region-2 from p16Pre36-NDM and pPp47. Furthermore, the structure of ΔTn*4352* is IS*26*–*aphA1a* in p16Pre36-NDM and ΔIS*26*–*aphA1a* in pPp47. The orientation of Tn*4352* in p16Pre36-NDM is direct, but reversed in pPp47and pPm60. The *aacC2*–*tmrB* region is present in plasmids pCTX-M3 and pU302L, is derived from transposon Tn*2* from the Tn*3*-family, and contains a IS*26* mobile element at its right-hand end (Partridge, 2011). The *aacC2* and *tmrB* genes account for aminoglycoside and tunicamycin resistance, respectively. The truncated *aacC2*–*tmrB* region in pPm60 is composed of an *aacC2*–*tmrB*–*orf192*–*orf228*–*orf1182* segment. The direction of the truncated *aacC2*–*tmrB* region is direct in pPm60, but reversed in pPp47. Owing to the insertion of a 28,064 bp exogenous region (with an unknown function), the truncated *aacC2*–*tmrB* region from pPp47 is segmented into two parts: ΔIS*Cfr1-*3′ exists in the MDR region-1, while a fragment from ΔIS*Cfr1-*5′ to the *aacC2* gene is embedded in the MDR region-2. Similarly, ΔTn*5563* is also located in the two MDR regions of pPp47. Tn*5563* was originally discovered in plasmid pRA2 from *Pseudomonas aeruginosa* (Yeo et al., 1998), and two segments of ΔTn*5563* in pPp47 are arranged as follows: the reverse segment (the *mer* operon and IRR) is present in MDR region-1 and the direct fragment (IRL and Δ*tnpR*) is present in MDR region-2 (Figures 1A, 2, 3).

### Other Accessory Modules Outside the MDR Region of pPrY2001-Like Plasmids

We found that Tn*6901* has a complete structure in pHFK418-NDM, but it is interrupted by insertion of the virulence-related region to generate two segments, ΔTn*6901-*5′ and ΔTn*6901-*3′. Tn*6901* is made up of an IRL–*tnpA*–*res*–*tnpR*–*frmB* (S-formylglutathione hydrolase)–*glo* (glyoxalase resistance)–*frmA* (S-glutathione dehydrogenase)–*frmR* (negative transcriptional regulator)–IRR structure in plasmid Rts1 from *Proteus vulgaris*, flanked by 5-bp DRs (Murata et al., 2002). Tn*6901* is inserted between *orf1528* and *orf942* in the backbone of pHFK418-NDM, bracketed by 5-bp DRs. pHFK418-NDM, a pPrY2001-like plasmid, is the only virulence gene-carrying plasmid, indicating that this plasmid can not only carry a large number of drug resistance genes, but also integrate virulence genes within it (Figure 1B).

It is known that *xerC* and *xerD* genes are site-specific recombinases in the lambda integrase family, where it was found that *xer*-mediated recombination events resulted in the transmission of resistance gene between plasmids and chromosomal locations (Merino et al., 2010). The *dfrA6*-*ereA* region is located downstream of the conjugal transfer region in p16Pre36-NDM, and has undergone *xer*-mediated recombination. The *dfrA6*-*ereA* region consists of *xerC*, *recD*, *xerD*, *dfrA6* (trimethoprim resistance), *ereA* (erythromycin resistance), and *dinB* (Supplementary Figure S2).

## Conclusion

The *bla*_NDM–__1_-harboring pHFK418-NDM plasmid, a pPrY2001-like plasmid group member, was first recovered from a clinical multidrug resistant *P. mirabilis* HFK418 isolate in China. Our data have revealed that the pHFK418-NDM plasmid contains two novel transpositions, Tn*6624* and Tn*6625*. Tn*6625*, a large composite transposon, has integrated a variety of mobile elements, such as the *bla*_NDM–__1_-carrying ΔTn*125*, *mph*(E)-harboring Tn*6624*, and In251. In251 was first identified from the above-mentioned clinical isolate, suggesting that it had been efficiently transferred from environmental organisms to clinical isolates. The pHFK418-NDM plasmid was found to have the ability for conjugal transfer, and to harbor a large numbers of resistance and virulence genes.

The pPrY2001-like plasmids described above harbor a wide variety of antimicrobial resistance genes, with the exception of p06-1619-1. Their relatively conserved backbones have integrated a great variety of accessory modules in the form of resistance genes, gene clusters, insertion sequences, transposons, and integrons, all of which enhance the diversification and evolution of the pPrY2001-like plasmids. Our findings augment our current understanding on the horizontal transfer of resistance genes and the genetic diversity and evolution of pPrY2001-like plasmids.

## Author Contributions

YZ, YT, and XZ conceived the study and designed the experimental procedures. DD, ZL, JF, NJ, and HZ performed the experiments. DD and ML analyzed the data. YZ, YT, XZ, BZ, and TZ contributed to reagents and materials. YZ, YT, and DD wrote the manuscript.

## Conflict of Interest Statement

The authors declare that the research was conducted in the absence of any commercial or financial relationships that could be construed as a potential conflict of interest.

## Footnotes

1 https://inkscape.org/en/

2 https://www.ncbi.nlm.nih.gov/WebSub/?form=history&session=new&tool=genbank
