# Supplementary material for: Characterization of a NDM-1- Encoding Plasmid pHFK418-NDM From a Clinical Proteus mirabilis Isolate Harboring Two Novel Transposons, Tn6624 and Tn6625
Source: Front Microbiol. 2019 Sep 4;10:2030. doi: 10.3389/fmicb.2019.02030 (PMC6737455; doi:10.3389/fmicb.2019.02030)
Supplement: Supplementary file 1 [file Data_Sheet_1.docx]

Supplementary Material

## Supplementary Figures

**Figure S1**

**
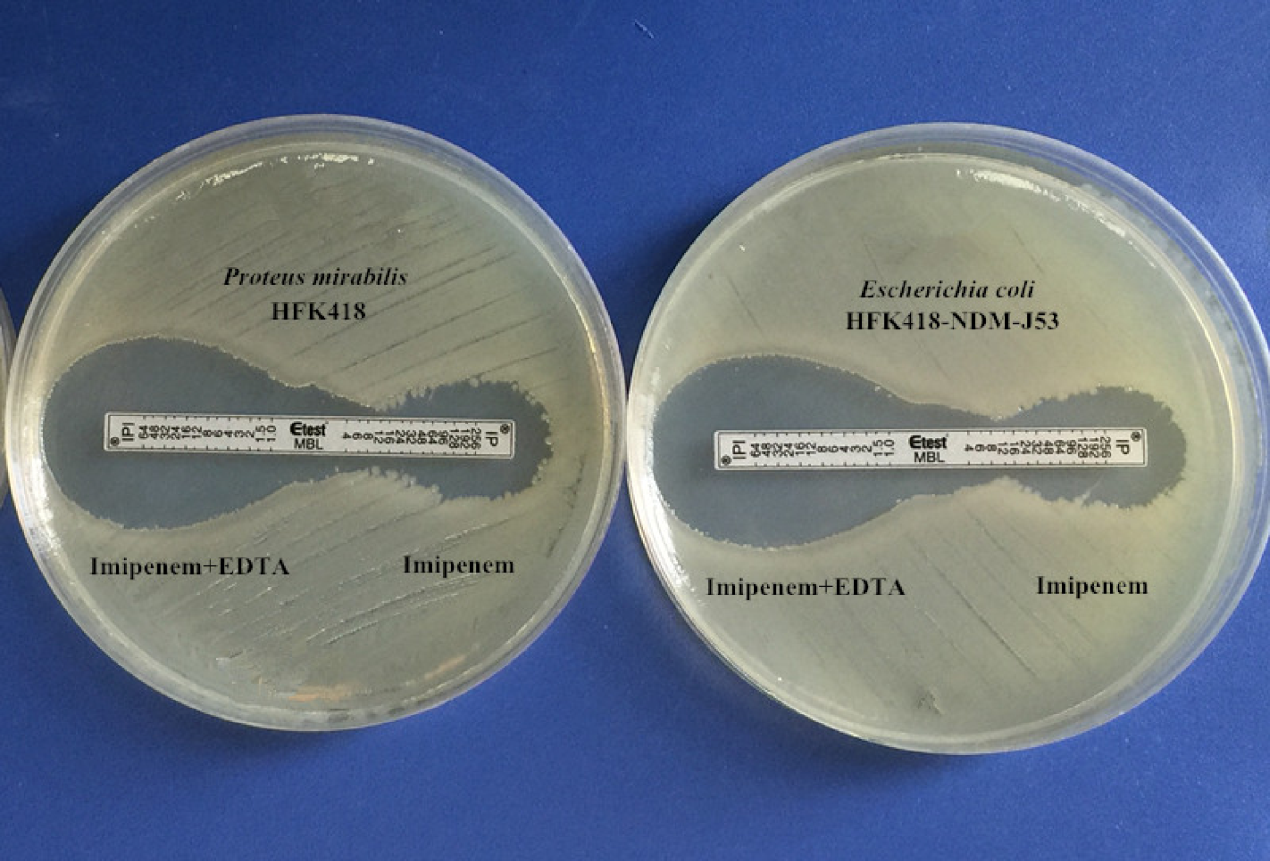
**

**Figure S1. Carbapenemase activity assay** **(imipenem–EDTA E-test)**

*P. mirabilis* HFK418 and *E. coli* J53Azi^R^ transconjugant strain HFK418-NDM-J53 were detected by imipenem–EDTA E-test.

**Figure S2:**


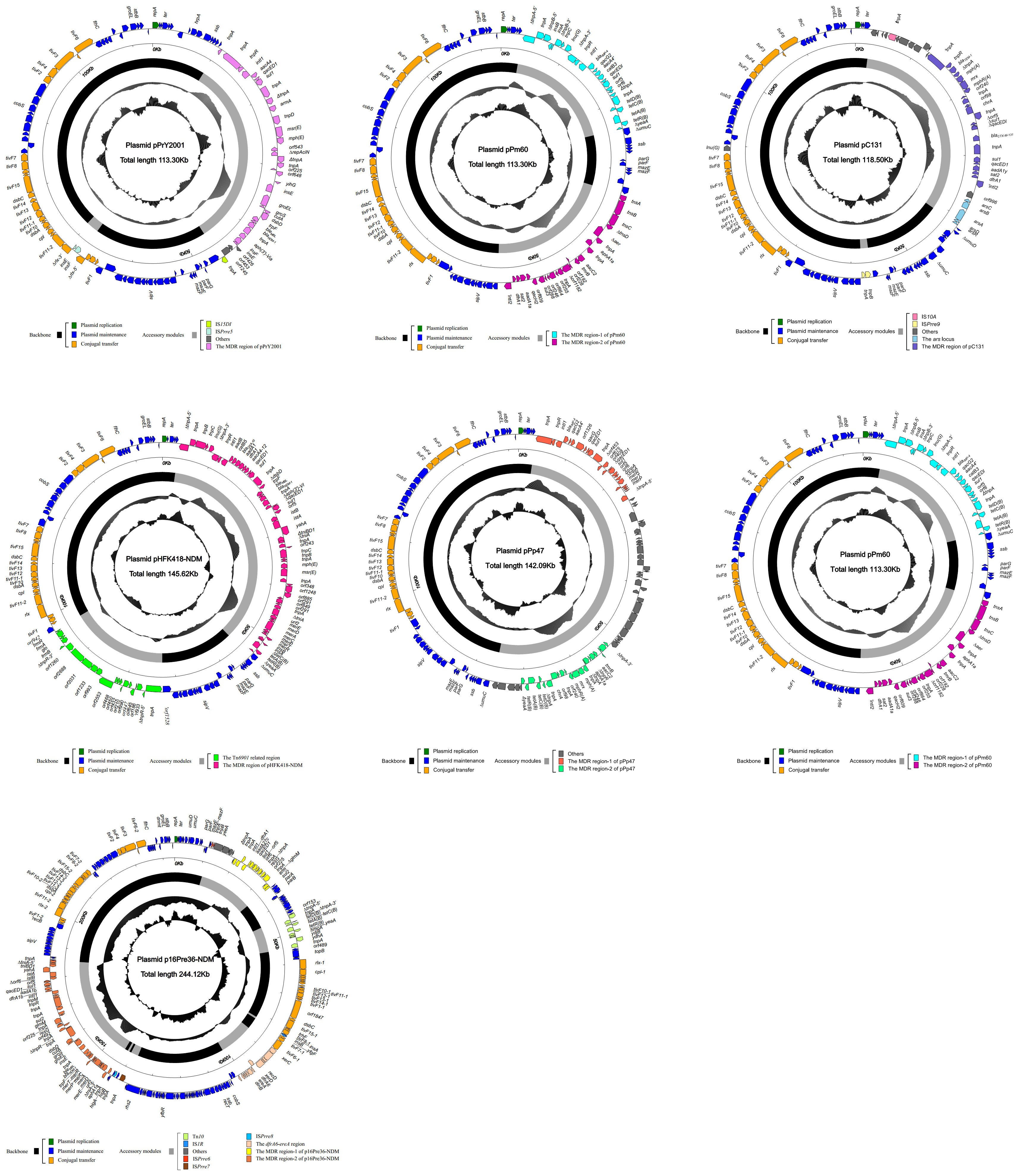


**Figure S2. Schematic maps of pHFK418-NDM and related plasmids.**

The seven plasmids pPrY2001 (KF295828), p06-1619-1 (KX832929), pC131 (KX774387), pHFK418-NDM (this study), pPp47 (MG516912), pPm60 (MG516911), and p16Pre36-NDM (KX832927) are included in the comparative analysis. Genes are denoted by arrows, and the backbone and accessory module regions are highlighted in black and color, respectively. The innermost circle presents GC-skew [(G-C)/(G+C)], with a window size of 500 bp and a step size of 20-bp. The next-to-innermost circle presents GC content.

**Figure S3:**


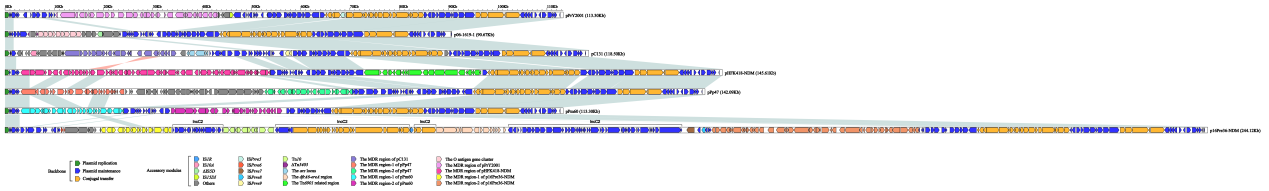


**Figure S3. Linear comparison of pHFK418-NDM with related plasmids.**

A linear comparison was carried out for the complete DNA sequences of pPrY2001 (KF295828), p06-1619-1 (KX832929), pC131 (KX774387), pHFK418-NDM (this study), pPp47 (MG516912), pPm60 (MG516911), and p16Pre36-NDM (KX832927). Genes are denoted by arrows. Genes, mobile elements and other features are colored based on function classification. Shading denotes regions of homology (>95% nucleotide identity).
